# Metal–Support Interaction Induced Electron Localization in Rationally Designed Metal Sites Anchored MXene Enables Boosted Electromagnetic Wave Attenuation

**DOI:** 10.1007/s40820-025-01819-9

**Published:** 2025-06-23

**Authors:** Xiao Wang, Gaolei Dong, Fei Pan, Cong Lin, Bin Yuan, Yang Yang, Wei Lu

**Affiliations:** 1 Shanghai Key Lab of D&A for Metal-Functional Materials, School of Materials Science and Engineering, Tongji University, Shanghai, 201804 People’s Republic of China; 2 College of Materials Science and Engineering, Fuzhou University, Fuzhou, 350108 People’s Republic of China

**Keywords:** Electron localization, Ti_3_C_2_T_x_-MXene, Microwave absorption, Metal–support interaction

## Abstract

The electron localization is considered as a promising approach to optimize electromagnetic waves (EMW) dissipation. However, it is still difficult to realize well-controlled electron localization and elucidate the related EMW loss mechanisms for current researches. In this study, a novel two-dimensional MXene (Ti_3_C_2_T_x_) nanosheet decorated with Ni nanoclusters (Ni-NC) system to construct an effective electron localization model based on electronic orbital structure is explored. Theoretical simulations and experimental results reveal that the metal–support interaction between Ni-NC and MXene disrupts symmetric electronic environments, leading to enhanced electron localization and dipole polarization. Additionally, Ni-NC generate a strong interfacial electric field, strengthening heterointerface interactions and promoting interfacial polarization. As a result, the optimized material achieves an exceptional reflection loss (RL_min_) of − 54 dB and a broad effective absorption bandwidth of 6.8 GHz. This study offers critical insights into the in-depth relationship between electron localization and EMW dissipation, providing a pathway for electron localization engineering in functional materials such as semiconductors, spintronics, and catalysis.

**Supplementary Information:**

The online version contains supplementary material available at 10.1007/s40820-025-01819-9.

## Highlights

- It is found that the reconstruction of the electronic environment and the optimization of the electromagnetic attenuation mechanism are achieved by constructing a model of metal–support interaction regulated electron localization.
- An excellent RL_min_ of − 54 dB and an ultra-wide effective absorption bandwidth of 6.8 GHz are obtained in the 2D MXene system.
- The microscopic pathway of “local field-driven carrier migration → interfacial electron rearrangement → dipole polarization enhancement” has been elucidated.

**Supplementary Information:**

The online version contains supplementary material available at 10.1007/s40820-025-01819-9.

## Graphical abstract

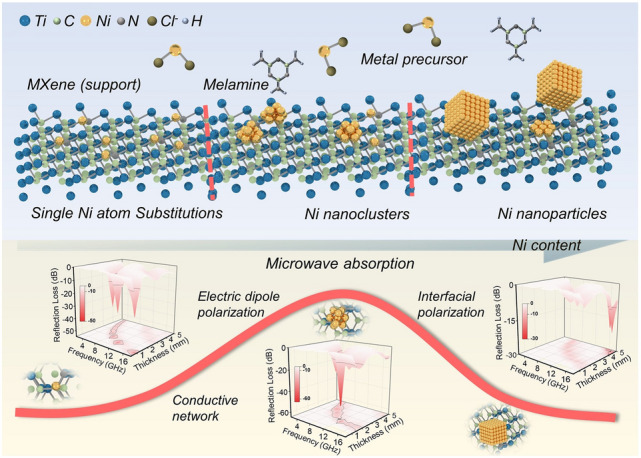

## Introduction

The rapid advancement of emerging technologies, such as the Internet of Things (IoT), artificial intelligence (AI), and the Metaverse, has led to increasingly complex electromagnetic pollution and interference, driving the exploration of high-performance electromagnetic waves (EMW) absorption materials [1–5]. Fundamentally, the electromagnetic response of macroscopic materials is governed by their microscopic structure, which is dictated by atomic and electronic arrangements [6–9]. When EMWs interact with a material, the local electronic environment determines the available energy states for electron excitation [10–12]. Variations in atomic configurations can induce changes in discrete energy levels or band structures within localized regions, affecting whether electrons can absorb EMW photons based on energy matching conditions [13–15]. Consequently, electron displacement within local environments induces diverse polarization mechanisms that critically govern EMW dissipation [16–18], with the efficiency and dominant nature of these polarization processes being strongly dependent on the degree of electron localization [19–24]. Electron localization is a phenomenon in which the movement of electrons in a material is limited and they are unable to diffuse freely over a long range [25–27]. This phenomenon is usually caused by disordered structures, strong correlation effects, or special geometrical arrangements, and has a profound effect on the electrical, magnetic, and optical properties of materials [28]. Although significant progress has been made in developing EMW absorption materials, the precise design and optimization of their performance remain hindered by an incomplete understanding of the relationship between the local electronic environment and electromagnetic properties, and the fundamental mechanisms by which electron localization modulates polarization for enhanced microwave absorption remain elusive, particularly at the atomic scale.

To regulate electron localization, researchers have explored structural design strategies such as atomic spacing modulation, molecular adsorption, and element doping [29]. However, achieving atomic-precision control over the electronic configurations of model systems persists as a fundamental challenge in materials design. Metal atoms, due to their abundant surface electrons, offer a promising route for tuning electron localization and, consequently, the surrounding electronic environment [25, 30]. In particular, constructing metal sites on material surfaces has emerged as an effective approach for finely modulating electron localization [26]. Among various candidates, transition metal sites with distinct d-orbital configurations have attracted significant interest [31]. Additionally, two-dimensional (2D) nanosheets, with their high surface-to-volume ratio and limited number of atomic layers, serve as an ideal model for studying the relationship between local electronic environments and electromagnetic properties at the atomic/molecular scale [32].

Recently, 2D transition metal carbides (MXenes), particularly Ti₃C₂Tₓ, have gained attention as EMW absorbers due to their unique metallic conductivity, high surface area, and tunable surface chemistry [33–35]. The 2D layered architecture of MXene maximizes the exposure of vacancy sites, while its abundant surface functional groups facilitate the electrostatic adsorption of metal precursors. Moreover, the defect-rich MXene surface not only efficiently captures metal atoms but also facilitates the self-reduction of metal precursors due to its high reactivity [31]. This anchoring of metal species provides an effective approach to control the electronic structure, charge transfer dynamics, and interfacial interactions, consequently modifying the local electronic environment [36, 37]. The characteristics of MXene-metal hybrids are predominantly governed by the metal–support interaction (MSI) effect, which alters energy band structures, electron transport behavior, and charge distribution [38–40]. MSI effect typically involves orbital hybridization and interfacial charge transfer at metal–support junctions, thereby altering the adsorption configurations of reaction intermediates and modulating energy barriers of elementary reaction steps. The MXene substrate offers a highly tunable ligand environment for supported metal species, enabling versatile MSI configurations [41]. This interaction facilitates the formation of electric dipoles and enhances interfacial polarization [42]. Strong MSI between MXene and anchored metal species can significantly tune the local electronic structure, promote electron orbital hybridization, and enhance charge transfer at the metal–MXene interface [43]. These effects collectively strengthen the dielectric dipolar polarization response in the microwave frequency range, making MSI engineering an effective strategy for achieving broadband EMW absorption [13, 44–46]. However, the fundamental physical mechanism underlying the influence of electron localization in MXene-metal systems on EMW dissipation remains largely unexplored. Therefore, precise control of the local electronic environment in MXene-metal composites is essential to unravel the mechanisms by which electron localization evolution impacts microwave absorption performance.

In this work, we achieve precise modulation of the local electronic environment through the controlled anchoring of Ni species on MXene nanosheets. Through the strategic anchoring of single atoms, sub-nanoclusters, and nanoparticles onto the MXene surface, we effectively engineer structural defects, modulate the electronic distribution, and promote spatial charge separation. The well-defined Ni sub-nanoclusters immobilized in the MXene matrix exhibit exceptional EMW absorption properties, achieving a minimum reflection loss (RL_min_) of − 54 dB and an effective absorption bandwidth (EAB) of 6.8 GHz at a thickness of 2 mm, attributed to polarization behavior induced by surface electronic localization. Radar cross section (RCS) and power loss density (PLD) simulations further confirm the strong electromagnetic attenuation capability. This strategic approach enables precise tuning of MXene’s electronic structure, enhancing its electromagnetic properties and presenting a promising pathway for surface electronic environment modulation in functional materials.

## Experimental Method

### Materials

The Ti_3_AlC_2_ (99%, 200 mesh) was purchased from Laizhou Kai Xi Ceramic Materials Co., Ltd. Concentrated hydrochloric acid (HCl, 12 M) and lithium fluoride (LiF (99.99%)) were obtained from Sinopharm Chemical Reagent Co., Ltd., Beijing, China. Nickel (II) chloride hexahydrate (NiCl_2_·6H_2_O) and melamine were purchased from Shanghai Titan Technology Co. LTD. The deionized water used in this experiment with a resistivity of 18.2 MΩ cm^−1^ was prepared through ion-exchange and filtration. All the chemicals were analytical grade and used without further purification.

### Preparation of Single/Few-Layer Ti_3_C_2_T_x_ Nanosheets

The Ti_3_C_2_T_x_ nanosheets containing abundant titanium vacancies were synthesized through a modified minimal intensive layer delamination (MILD) protocol. 2 g LiF was incrementally introduced to 40 mL HCl (12 M) and the mixture underwent sustained stirring for 10 min to achieve complete dissolution, yielding a homogeneous etchant precursor solution. The etching reaction was initiated by gradual addition of 1 g Ti_3_AlC_2_ MAX phase precursor to the etchant. The reaction system was subsequently heated to 50 °C and maintained isothermal for 24 h to ensure complete Al layer removal. The multilayer Ti_3_C_2_T_x_ product was deionized with deionized water to remove excess LiF and other impurities, as well as precipitates that can be obtained by washing until the pH is about 6. The purified multilayer product was homogenously dispersed in deionized water and exfoliated via argon-protected ultrasonication under ice-bath cooling for 30 min. The resulting colloidal suspension was then centrifugally fractionated at 3500 r min^−1^ for 60 min, during which the dark green supernatant enriched with monolayer/few-layer nanosheets was carefully decanted for further characterization. The nanosheet dispersion was rapidly frozen in liquid nitrogen and stored at − 18 °C. For powder characterization, frozen samples were lyophilized over 48 h to obtain Ti_3_C_2_T_x_.

### Preparation of Ni-MX

Initially, 50 mg Ti_3_C_2_T_x_ was dispersed in 15 mL deionized water through ultrasonication (30 min), followed by magnetic stirring (30 min) to achieve colloidal stability. Controlled molar quantities of NiCl_2_·2H₂O (8.4 × 10^−3^, 2.5 × 10^−2^, 4.2 × 10^−2^, 6.3 × 10^−2^, and 8.4 × 10^−2^ mmol) were incrementally introduced under continuous agitation (1 h), succeeded by the introduction of 100 mg melamine and stirred for 1 h. Then, ultrasound was performed in an ice bath for 30 min before freeze-drying (− 80 °C, 48 h) to obtain precursor powders. Thermal treatment was conducted in a tube furnace under Ar flow with programmed heating (500 °C, 2 h). The obtained sample was labeled Ni1-MX, Ni2-MX, Ni3-MX, Ni4-MX, and Ni5-MX, respectively.

### Preparation of MX

The same prepared procedure of Ni-MX was conducted for the synthesis of MX without the addition of metal precursors.

### Characterization

We used a DX-2700 X-ray diffractometer (Cu-Kα radiation, *λ* = 1.54 Å) to obtain the X-ray diffraction (XRD) patterns. The microstructures of the samples were observed using transmission electron microscopy (TEM, H-800). To determine the chemical states and surface components of the samples, we utilized X-ray photoelectron spectroscopy (XPS) on a Thermo Scientific K-Alpha spectrometer. The room magnetic temperature hysteresis loops were acquired with a vibrating sample magnetometer (VSM, manufactured by Lakeshore, Inc.). Using the coaxial-line theory, we measured the related electromagnetic wave (EMW) parameters of samples in the frequency range of 2–18 GHz on a vector network analyzer (VNA, 3672B-S, Ceyear). Specifically, the sample was homogeneously blended with molten paraffin wax and then pressed into a ring-shaped mold (inner diameter Φ_in_ = 3.04 mm, outer diameter Φ_out_ = 7 mm) for testing purposes. The mass percentage of the sample in the paraffin mixture was 5 wt%.

### Radar Cross Section (RCS) Simulation Protocol

The RCS characteristics of the EMW absorber were investigated using CST software based on far-field scattering analysis. A multilayered simulation model was constructed, comprising a composite absorber/paraffin layer (5 wt% FL) and a perfect electric conductor (PEC) reference layer, with the latter serving as the baseline for quantifying RCS reduction performance. Both layers were geometrically constrained to 200 × 200 mm^2^ planar dimensions, with optimized thicknesses of 2.0 mm for the absorber/paraffin composite and 5.0 mm for the PEC substrate. The computational domain employed a plane-wave excitation scheme propagating along the negative x-axis with z-axis polarized electric field components. Boundary conditions were configured to approximate free-space propagation, and simulations were centered at 12.2 GHz to align with X-band radar applications. The time-domain solver implemented a finite integration technique (FIT) to calculate the far-field RCS values, governed by the fundamental radar equation:

$$\sigma \left( {{\text{dB}}\,m^{2} } \right) = 10\,\log \left[ {\frac{4\pi S}{{\lambda^{2} }}\left| {\frac{{E_{{\text{S}}} }}{{E_{{\text{i}}} }}} \right|^{2} } \right]$$

where *S* is the area of the layer, *λ* is the length of the incident EMW, and *E*_s_ and *E*_i_ are the electric field intensity of transmitting waves and receiving waves, respectively.

### E-filed and Power Loss Density Simulation

The E-filed and power loss density simulation of EMW absorber was used CST software based on far-field response. In this simulation, the constructed model consists of the absorber, the length and width of each layer were set as 5 mm and the thickness of the absorber layer was set as 2 mm. The frequency was set at 12.2 GHz. Perfectly matched conditions were imposed on the air domain to eliminate interference from the reflected waves. Two ports (port 1 and port 2) were established in the air domain to calculate the S-parameters. Port 1 was activated with 1 W input power in electric field mode.

### Density Functional Theory Methods

The first-principles calculations were performed based on density functional theory (DFT). The electron–ion interactions were described using the projector augmented wave (PAW) method, and the exchange–correlation functional employed the generalized gradient approximation (GGA) in the Perdew–Burke–Ernzerhof (PBE) formulation. A plane-wave cutoff energy of 500 eV was adopted, and the Brillouin zone was sampled using a 3 × 3 × 1 Monkhorst–Pack k-point mesh. The convergence criteria for electronic self-consistent calculations and ionic relaxations were set to 10–5 eV and 0.01 eV Å^−1^, respectively. A 4 × 4 × 1 supercell model was constructed with a vacuum layer of ~ 16 Å along the *z*-direction to eliminate spurious periodic interactions. Three doping configurations (single-atom, nanoclusters, and nanocrystalline) were investigated. During structural optimization via the conjugate gradient algorithm, the bottom two atomic layers were fixed to mimic bulk substrates, while the upper layers were fully relaxed. Differential charge density, density of states (DOS), and band structure analyses were conducted to elucidate the electronic interplay in these systems.

## Result and Discussion

### Characterization of Ni-MX

**Fig. 1:**
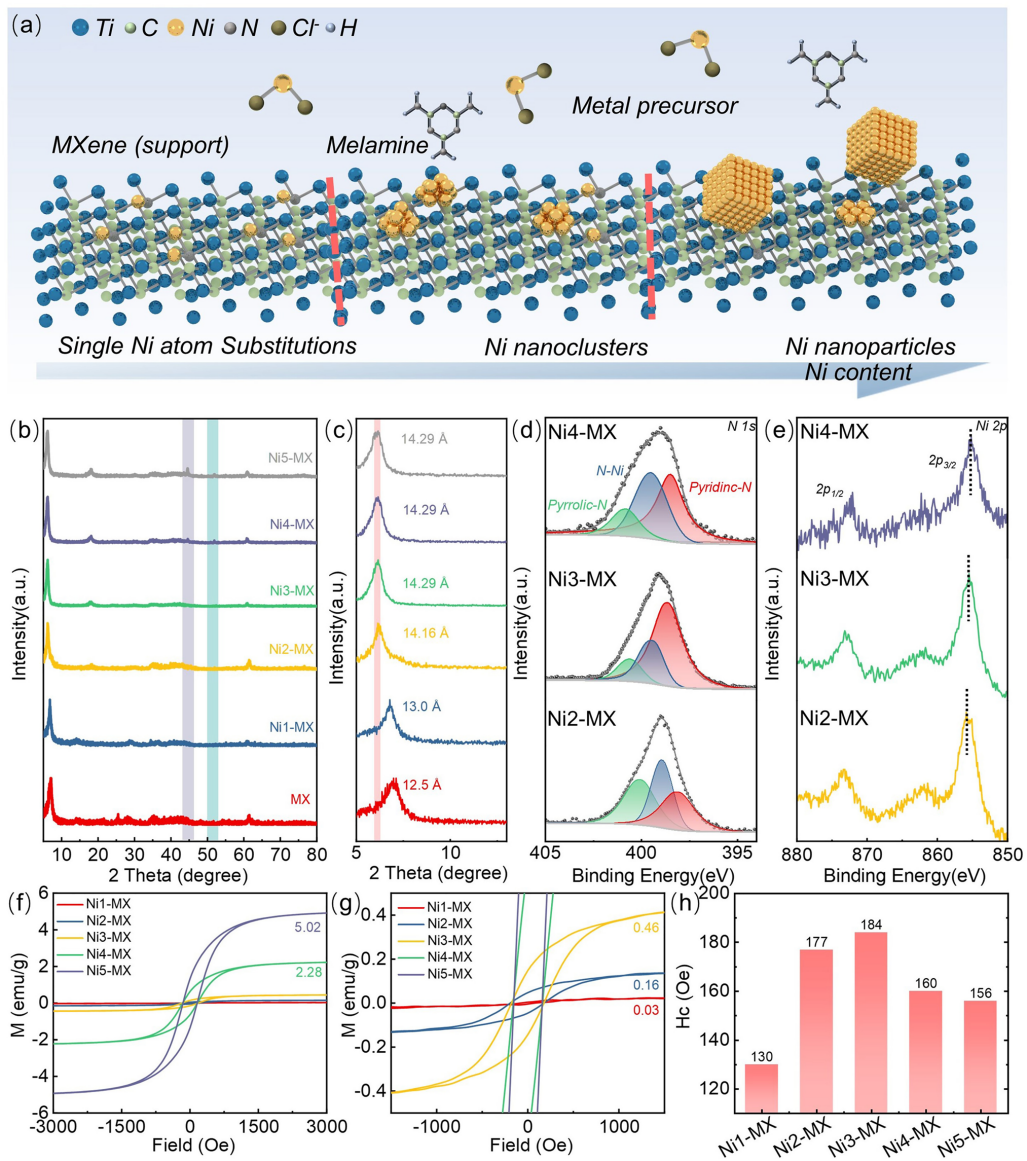
Structural characterization of Ni-MX materials. **a** Schematic representation of the fabrication process for Ni-MX. **b** XRD pattern of Ni-MX. **c** Comparison of XRD patterns for different Ni-MX samples. **d** XPS survey spectrum of nitrogen in Ni-MX. **e** XPS survey spectrum of nickel in Ni-MX. **f** VSM survey results for Ni-MX, illustrating magnetic properties. **g** Magnetic hysteresis loop of Ni-MX. **h** Coercivity (*H*_c_) values of Ni-MX

The Ti_3_C_2_T_x_ MXene is synthesized through the selective etching of the aluminum (Al) layer from the layered ternary transition metal carbide Ti_3_AlC_2_ MAX, employing hydrochloric acid (HCl) and lithium fluoride (LiF) [47]. During this etching process, the dissolution of the Al layer results in the exfoliation of Ti_3_AlC_2_ into either monolayer or multilayer ultrathin Ti_3_C_2_T_x_ Mxene [48]. This treatment creates a rich array of terminal groups, denoted as “T” (including O, OH, and F), on the surface of the MXene, facilitating the adsorption of metal precursors [49]. Notably, the etching process also removes some adjacent titanium (Ti) atoms, thereby introducing Ti vacancy defects on the surface of Ti_3_C_2_T_x_ MXene, which enables the possibility of heterogeneous substitution [50]. Subsequently, nickel (II) chloride hexahydrate (NiCl_2_·6H_2_O) and melamine are introduced into the Ti_3_C_2_T_x_ suspension during continuous stirring, promoting the self-reduction of Ni^2+^. Through precise thermal treatment at 500 °C, the precursors undergo controlled transformation into well-dispersed Ni–N–C coordination structures with atomic-scale separation. By adjusting the quantity of precursors, scalable preparation is achieved, resulting in the synthesis of Ti_3_C_2_T_x_ MXene modified with single atoms, sub-nanometer clusters, and nanoparticles, which is illustrated in Fig. 1a.

Utilizing XRD (Fig. 1b, c), we further characterized the various samples obtained. The absence of the (104) diffraction peak for Ti_3_AlC_2_ in the products indicates successful removal of the aluminum layer [51]. In the XRD spectra, the samples Ni1-MX, Ni2-MX, and Ni3-MX lack peaks corresponding to nickel-related crystalline phases, suggesting that the nickel species are well dispersed. With increase in introduction of metal ions, well-defined nickel peaks gradually appeared, indicating the formation of metallic nickel nanoparticles. As illustrated, the low-angle XRD pattern of few-layered Ti_3_C_2_T_x_ displays a prominent (002) characteristic peak at 6.98°. Following the modification of Ti_3_C_2_T_x_ with either single atoms or clusters, a shift of the (002) peak to a lower angle was observed, indicating an expansion of the interplanar spacing from 12.5 to 14.16 Å. After the formation of sub-nanometer clusters, the interplanar spacing of Ti_3_C_2_T_x_ no longer exhibited any significant shifts, thereby confirming the substantial doping of nickel sub-nanoclusters at the surface defects of MXene.

XPS (Figs. 1d, e, S1–S2) was employed to analyze the chemical elements and their valence states present within the samples. The XPS spectrum reveals the presence of carbon (C), nitrogen (N), oxygen (O), titanium (Ti) and nickel (Ni) elements. High-resolution N 1*s* spectra exhibit peaks that can be deconvoluted into contributions from pyridinic nitrogen (398.7 eV) and pyrrolic nitrogen (400.4 eV), confirming the successful doping of nitrogen heteroatoms into the carbon lattice. Furthermore, a new peak associated with Ni–N bonding appears in the N 1*s* spectrum, indicating an interaction between metallic atoms and the MXene matrix via the Ni–N–C pathway. Analysis of the oxidation state of Ni atoms is conducted through the Ni 2*p* spectrum, which presents two peaks at 873.7 and 855.1 eV, corresponding to the 2*p*_1/2_ and 2*p*_3/2_ levels, respectively. The position of the Ni 2*p*_3/2_ peak lies between those of Ni^0^ (853.0 eV) and Ni^2+^ (856.5 eV), confirming that the introduced nickel species are reduced by reactive titanium vacancies in the Ti_3_C_2_T_x_ MXene nanosheets, resulting in oxidation states of Ni ranging from 0 to + 2. Notably, the binding energies of Ni species in Ni-SA/MX are shifted in a higher direction compared to those in Ni-NP/MX. The higher valence state of the Ni species in Ni-SA/MX implies a stronger interaction with the support, with more surface charge being transferred from the Ni atom to the support through the Ni–N bonds [36].

The magnetic properties of the composite materials were investigated by measuring the magnetization curves under an applied magnetic field (M-H) (Fig. 1f–h). Distinct hysteresis loops are observed at room temperature, indicating typical ferromagnetic behavior. In contrast, the Ni1-MX composite does not exhibit pronounced hysteresis, which can be attributed to its weak magnetic characteristics. The saturation magnetization exhibits a gradual increase corresponding to the morphological transition of Ni species from isolated atoms to clusters and then to nanoparticles. Notably, the higher saturation magnetization observed in Ni3-MX, Ni4-MX, and Ni5-MX at room temperature primarily arises from atomic clusters and NPs. Although the Ni species are embedded as isolated atoms within the MXene matrix, the Ni2-MX composite still demonstrates characteristic magnetic features at room temperature [52]. DFT calculations reveal that the spatially isolated Ni atoms embedded within the MXene matrix give rise to magnetism in Ni2-MX and Ni3-MX, exhibiting high occupancy states around the Fermi level. This ensures magnetic behavior at room temperature, with the high occupancy states originating from the synergistic contributions of d electrons from metallic Ni and p electrons from the matrix, indicating strong orbital hybridization between the d and p orbitals [53].

**Fig. 2:**
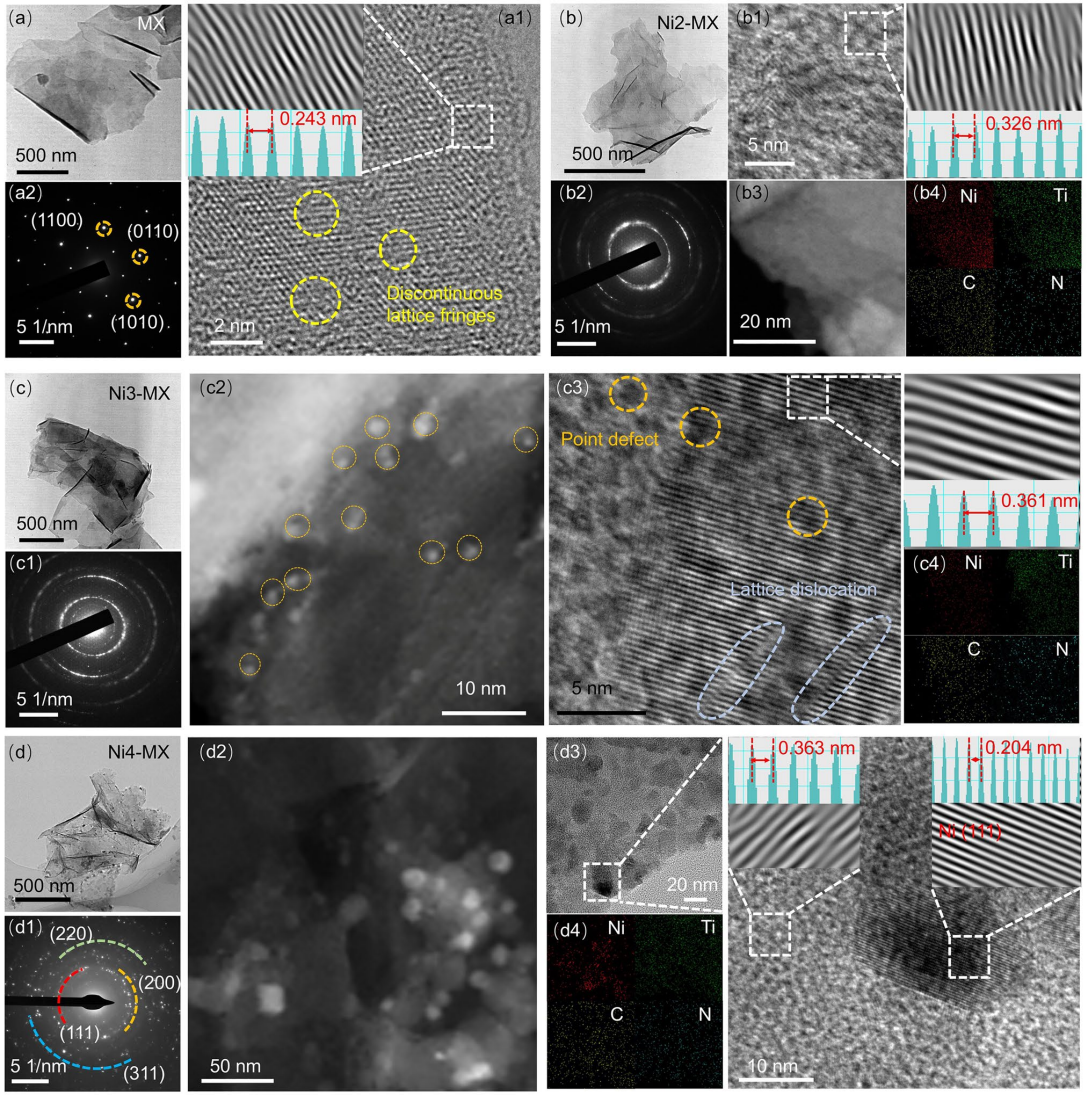
TEM characterization of MX and Ni-MX composites. **a** TEM image of MX. **a1** HRTEM image of MX. **a2** SAED pattern of MX. **b** TEM image of Ni2-MX. **b1** HRTEM image of Ni2-MX. **b2** SAED pattern of Ni2-MX. **b3** HAADF-STEM image of Ni2-MX. **b4** Elemental mapping of Ni2-MX. **c** TEM image of Ni3-MX. **c1** SAED pattern of Ni3-MX. **c2** HAADF-STEM image of Ni3-MX. **c3** HRTEM image of Ni3-MX**. c4** Elemental mapping of Ni3-MX. **d** TEM image of Ni4-MX. **d1** SAED pattern of Ni4-MX. **d2** HAADF-STEM image of Ni4-MX. **d3** HRTEM image of Ni4-MX. **d4** Elemental mapping of Ni4-MX

The morphology and microstructure of the samples were characterized using TEM (Figs. 2, S3), revealing single or few-layered structures of Ni-MX, suggesting that the introduction of trace amounts of guest metal ions has a negligible effect on morphology (Fig. 2b–d). High-angle annular dark field scanning transmission electron microscopy (HAADF-STEM) (Fig. 2b3, c2, d2) and energy-dispersive X-ray spectroscopy (EDX) elemental mapping (Fig. 2b4, c4, d4) are employed for a more detailed investigation into the distribution of nickel on Ti_3_C_2_Tx MXene. Figure 2a is the TEM of MX, showing the successful preparation of 2D MXene material with good crystallinity which is shown in Fig. 2a1, a2. Figure 2b is the TEM of Ni2-MX, where the introduction of Ni atoms breaks the crystallinity of the MXene itself and expands the interplanar spacing (Fig. 2b1, b2). EDX mapping confirmed that the nickel atoms are uniformly dispersed across the MXene surface (Fig. 2b4). As the concentration of the introduced Ni species increased, the Ni-NC-anchored MXene (Ni3-MX) is shown in Fig. 2c. As illustrated in Fig. 2c2, uniformly sized sub-nanometer nickel clusters are consistently anchored on the surface of the MXene and the size distribution diagram is shown in Fig. S4. Furthermore, with an increased concentration of metal ions, well-crystallized nanoparticles with a lattice spacing of 0.204 nm anchored on the surface of MXene are observed (Fig. 2d), corresponding to the (111) crystallographic plane of metallic nickel (Fig. 2d1, d3). Furthermore, the matrix’s interplanar spacing exhibits a proportional increase with the doped metal species’ size, in agreement with XRD results.

### EMW Absorption Performance Modulation

**Fig. 3:**
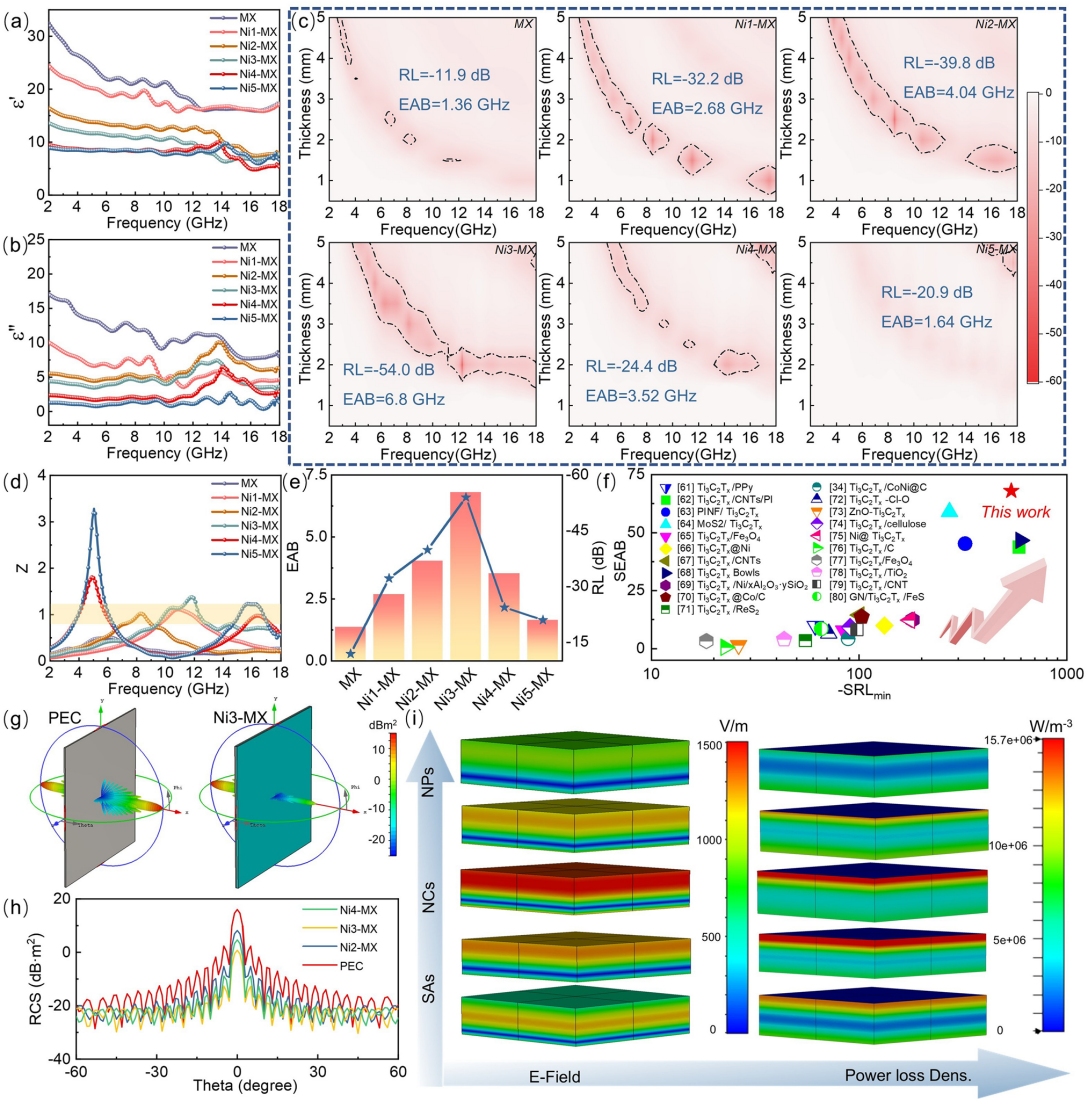
Electromagnetic wave absorption performance of Ni-MX composites. **a** Real ($\varepsilon^{\prime}$) and **b** imaginary ($\varepsilon^{\prime\prime}$) parts of permittivity. **c** 2D reflection loss (RL) performance **d** Z value of Ni-MX. **e** Comparison of RL_min_ and EAB_max_ among samples. **f** Comparison of SEAB and − SRL_min_ between Ni3-MX and typical MXene-based EMW absorbers. **g** 3D radar wave scattering signals of samples. **h** Simulated radar cross section (RCS) curves. **i** Schematic representation of E-field distribution and power loss density at 12 GHz for Ni-MX

The above results indicate that the uniquely structured two-dimensional layered material, composed of differently dispersed Ni single atoms, nanoclusters, and nanoparticles, may have varying effects on EMW absorption properties. A systematic analysis of the electromagnetic parameters of the samples was conducted, focusing on the complex permittivity $\left( {\varepsilon_{r} = \varepsilon^{\prime} - j\varepsilon^{\prime\prime}} \right)$ and complex permeability ($\mu_{r} = \mu^{\prime} - j\mu^{\prime\prime}$) to elucidate the intrinsic electromagnetic response mechanisms [54]. As illustrated in Fig. 3a, b, the values of $\varepsilon^{\prime}$ and $\varepsilon^{\prime\prime}$ for MXene fluctuate within the ranges of 32.5–17.3 and 17.08–8.35, respectively. Upon embedding Ni species into the MXene matrix in the form of isolated atoms, the values for Ni1-MX are recorded at 24.5–17.1 and 10.06–4.7. For Ni2-MX, the corresponding values of $\varepsilon^{\prime}$ and $\varepsilon^{\prime\prime}$ are 16.46–7.8 and 5.53–6.25, respectively. This decline can be attributed to the interactions between the isolated Ni atoms and the MXene substrate. The values for Ni3-MX further decrease to 13.61–7.6 and 4.39–3.59. For Ni4-MX and Ni5-MX, the values of $\varepsilon^{\prime}$ and $\varepsilon^{\prime\prime}$ are observed at 9.48–5.3 and 8.89–6.68, as well as 2.38–2.82 and 1.28–1.32, respectively. Given the relatively weak magnetic loss capabilities of the designed system, $\mu^{\prime}$ and $\mu^{\prime\prime}$ oscillate around 1 and 0, respectively (as illustrated in Fig. S5). Throughout the measured frequency range, the tangent of the dielectric loss is greater than that of the magnetic loss (as depicted in Fig. S6), indicating that dielectric loss predominates the dissipation mechanism [55]. This dissipation mechanism comprises dielectric polarization caused by polarization relaxation and conduction losses due to electron migration. While dielectric loss plays a significant role in electromagnetic wave absorption, the magnetic response remains equally important to consider [56]. The coordination of Ni atoms and clusters with N atoms embedded within the MXene matrix effectively activates the sample’s saturation magnetization, which can induce local magnetic moments, thereby enhancing the electromagnetic wave absorption performance [57].

The transmission line theory is utilized to assess the EMW absorption capacity of the samples. The performance of these EMW absorbers is evaluated based on the minimum reflection loss (RL) and effective absorption bandwidth (EAB), where RL ≤  − 10 dB defines the frequency range of effective absorption [58]. As shown in Fig. 3c, the MXene matrix exhibits relatively weak absorption properties, with a minimum RL of − 11.9 dB and a maximum EAB of 1.36 GHz at a thickness of 2.5 mm. With the introduction of Ni single atoms, Ni1-MX demonstrates a moderate improvement in RL, reaching − 32.2 dB at an optimized thickness of 1.5 mm, alongside an EAB of 2.68 GHz. In the case of Ni2-MX, the RL further decreases to − 39.85 dB, while the EAB expands to 4.04 GHz at the same thickness of 2.5 mm. Notably, Ni3-MX exhibits the most remarkable enhancement, achieving a minimum RL of − 54 dB and an EAB of 6.8 GHz at a reduced thickness of 2 mm. However, a further increase in Ni content results in performance deterioration, with Ni4-MX displaying an RL of − 24.4 dB and an EAB of 3.52 GHz. Similarly, Ni5-MX experiences an even greater decline, with an RL of − 20.97 dB and a significantly reduced EAB of 1.64 GHz (Fig. 3e).

Among the synthesized absorbers, Ni3-MX exhibits the most outstanding EMW absorption properties, highlighting the crucial role of Ni nanocluster coordination with N/C atoms in optimizing electromagnetic wave attenuation. Impedance matching (*Z*, |*Z*_in_/*Z*_0_|) and the attenuation constant (*α*) are two fundamental factors influencing electromagnetic response [5, 9]. An *Z* value closer to 1 suggests superior impedance matching, allowing electromagnetic waves to penetrate the absorber more effectively [59, 60]. As shown in Fig. 3d, the MXene matrix exhibits significant impedance mismatch, while the introduction of Ni species markedly improves impedance matching in Ni3-MX. The *α*–*f* relationship curves for the above materials are given in Fig. S7a, where the range of *α* values for MX is much larger than that of the doping products. It can be seen that the excessive attenuation ability and the degree of impedance mismatch are the main reasons for the poor wave absorption performance of the MXene matrix material. For Ni-MX materials, the intervention of Ni species mainly serves to optimize the interfacial impedance and reduce the surface skinning current, while the high dielectric of the matrix serves to enhance the overall attenuation capability. The *α*-value gradually decreases with the increase of Ni species size. From the above results, it can be seen that the excellent impedance matching characteristics and suitable attenuation ability are important factors to promote the optimal wave-absorbing performance of Ni3-MX samples at ultrathin thicknesses [22, 23]. In addition, as the thickness increases, the absorption peak shifts to lower frequencies, which can be explained by the quarter-wavelength matching model (Eq. S12) [20]. When dm and *f*_m_ satisfy the above equations, the incident and reflected waves will emit an interference effect, leading to the dissipation of EMW energy. In Fig. S9, the matching thickness agrees with the calculated thickness, indicating that the experimental results confirm the quarter-wavelength model. As a result, Ni3-MX demonstrates excellent absorption performance, further supported by its superior − SRLmin values compared to previously reported MXene-based absorbers, indicating its strong potential for advanced EMW absorption applications (Fig. 3f) [34, 61–80].

To further evaluate its real-world applicability, radar cross section (RCS) simulations were performed. As illustrated in Figs. 3g and S10, a pure perfect electric conductor (PEC) exhibits a strong RCS reflection signal. When coated with an absorbing layer, the PEC/absorber configuration significantly reduces the RCS signal, with Ni3-MX demonstrating the most pronounced reduction. Across a wide angular range (− 60° to 60°), Ni3-MX exhibits superior RCS suppression. At 0°, it achieves a maximum RCS reduction of 17 dB m^2^, confirming its strong EM attenuation capability, which is crucial for applications in extreme and complex environments (Fig. 3h). Furthermore, surface current density analysis provides insights into the impedance matching behavior, with a decrease in red regions indicating improved penetration of incident EM waves. The electric field (E-field) distribution and power loss density (PLD) at 12.2 GHz (Fig. 3i) reveal that, upon vertical EM wave incidence, the E-field and power losses are primarily concentrated in the upper-middle region of the absorber, leading to an extended propagation path and enhanced energy dissipation. Notably, Ni3-MX exhibits the highest PLD among all samples, effectively converting and dissipating greater amounts of EM energy. These findings further emphasize the exceptional EM response characteristics of Ni sub-nanocluster-modified absorbers.

### EMW Absorption Mechanisms

**Fig. 4:**
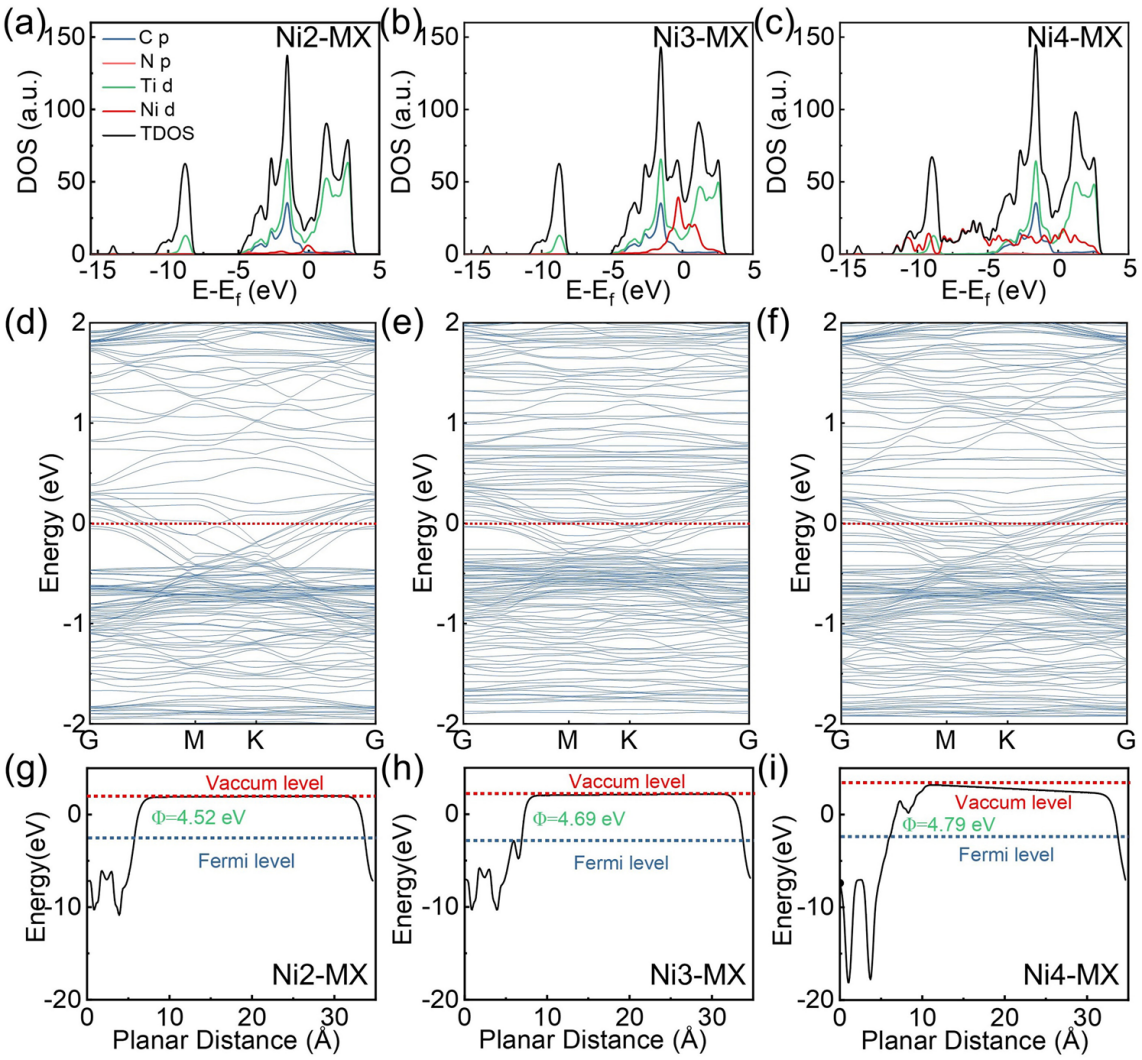
Electronic configuration of the Ni-MX composites. **a**–**c** Density of states. **d**–**f** Energy band structures. **g**–**i** Work function analysis of Ni2-MX, Ni3-MX, and Ni4-MX

In order to comprehensively investigate the effect of various nickel microstructures on causing changes in EMW absorption properties, DFT calculations were carried out. The DOS distribution curves (Fig. 4a) and band structure analysis (Fig. 4b–d) show that all DOS curves intersect the Fermi level, confirming the metallic nature of the materials. Notably, the total DOS at the Fermi level is highest for Ni3-MX, signifying more intense electron transitions, which is further validated by the corresponding charge distribution (Fig. S11a). The d-band center of Ti in Ni3-MX is positioned closer to the Fermi energy level, suggesting greater electron adsorption capability and reinforcing its enhanced dipole polarization behavior (Fig. S11b). The work function, which represents the minimum energy required for electron emission from the material’s surface, provides additional insights into interfacial charge transfer mechanisms (Fig. 4e–g). The differences in work functions between these three models, indicating the presence of different degree of interface polarization in Ni-MX. As shown in Fig. S12, the work function of the MXene matrix is 4.5 eV, and that of metallic Ni (111) is 5.1 eV. Consequently, when MXene and Ni species come into contact, electron migration occurs from Ni to MXene until a dynamic equilibrium is reached, generating strong interfacial polarization.

**Fig. 5:**
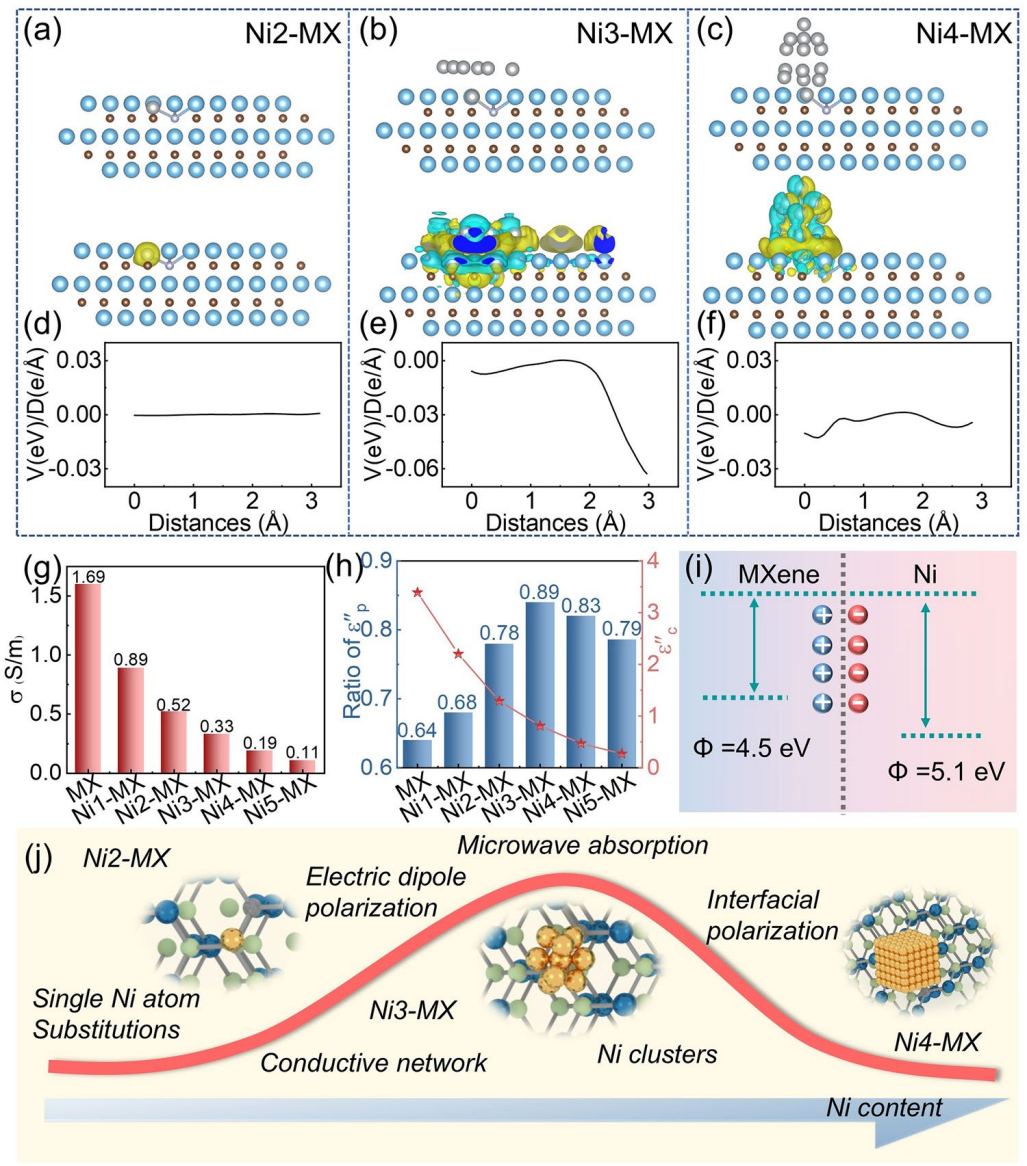
Mechanisms of EMW absorption in Ni-MX composites. **a**–**f** Differential charge density and line profile of interatomic potential/density. **g** σ of the Ni-MX. **h** Calculated conductive loss ($\varepsilon_{c}^{{^{\prime\prime}}}$) and the ratio of polarization loss ($\varepsilon_{p}^{{^{\prime\prime}}}$). **i** Electron migration behavior at Ni-MX heterogeneous interfaces. **j** Proposed EMW absorption mechanisms in Ni-MX composites

Differential charge density (Fig. 5a–c) reveals that while the charge distribution in Ni2-MX is relatively dispersed, it facilitates the formation of efficient electronic transport pathways. In contrast, the charge cloud complexity in Ni3-MX suggests potential restrictions on long-range electron mobility. The presence of cyan clusters represents significant local charge redistribution, which promotes electron localization. This phenomenon not only facilitates interfacial polarization but also enhances electron–phonon interactions, leading to increased electron scattering and reduced overall conductivity. Additionally, the electronic coupling interactions between Ni nanoclusters and MXene induce an asymmetric electronic environment. The resulting reconfigured electron distribution acts as a permanent dipole, enhancing dipole relaxation under alternating electromagnetic fields and thereby improving the response to EMW. For Ni4-MX, the periodic arrangement of Ni nanoparticles leads to stronger electron scattering. The formation of charge layers at the top and bottom of the structure generates potential barriers that further impede electron transport. And the line profile in Fig. 5d–f is the interatomic potential/density between the anchored Ni atom and the neighboring Ti atom, further confirming the more pronounced electron localization phenomenon observed in Ni3-MX. These DFT results confirm that surface microstructure modulation significantly influences the electrical properties of MXene, ultimately impacting its EM performance.

Electromagnetic energy dissipation arises from two primary charge behaviors: conduction loss ($\varepsilon_{c}^{{^{\prime\prime}}}$) and polarization loss ($\varepsilon_{p}^{{^{\prime\prime}}}$). The $\varepsilon^{\prime\prime}$ curve exhibits significant fluctuations across the frequency spectrum, indicative of multiple dielectric polarization relaxation processes [59, 81]. These variations can be attributed to the presence of grain boundaries, impurity atoms, or structural defects [82]. According to Debye theory, the Cole–Cole plot is used to analyze dielectric polarization, where each semicircle represents a distinct polarization relaxation process [83]. As shown in Fig. S13, the Cole–Cole plot of MXene consists of several irregular semicircles along with a linear segment, while Ni3-MX exhibits multiple semicircles, suggesting a stronger dielectric polarization response and a more pronounced contribution from conductive loss. Furthermore, the Cole–Cole semicircle demonstrates a distinct correlation with the characteristic frequencies of dielectric constant fluctuations. A representative analysis of Ni3-MX reveals three most distinct semicircular features emerging within the 6–16 GHz range, centered at approximately 7.5, 11.8, and 13.9 GHz, respectively (Fig. S14). Notably, these characteristic frequencies exhibit exact correspondence to the peak positions of dielectric constant fluctuations observed in Fig. 3a, b. This alignment strongly suggests that polarization relaxation mechanisms underlie the variations in electromagnetic parameters at these specific frequencies. Other samples also display multiple semicircles and linear features in their respective Cole–Cole diagrams, indicating that both conductive and polarization losses play a significant role in electromagnetic energy dissipation.

To further elucidate the mechanisms governing the dielectric response, we quantified the contributions of conduction and polarization losses. The measured electrical conductivities of MX, Ni1-MX, Ni2-MX, Ni3-MX, Ni4-MX, and Ni5-MX were 1.37, 0.89, 0.52, 0.33, 0.19, and 0.11 S m^−1^, respectively (Fig. 5g). Notably, Ni2-MX exhibits the most homogeneous electronic distribution, facilitating efficient electron transport. Although the structure of Ni3-MX appears more disordered, it retains a degree of electronic mobility. In contrast, Ni4-MX introduces an increased number of grain boundaries and defects, which hinder electron transport, leading to lower conductivity. To isolate the contributions of $\varepsilon_{c}^{{^{\prime\prime}}}$ and $\varepsilon_{p}^{{^{\prime\prime}}}$, we employed a parallel/series resistance–capacitance circuit model and applied nonlinear fitting using the least-squares method. Conductive loss was evaluated based on electronic conductivity, where $\varepsilon_{c}^{{^{\prime\prime}}}$ is directly proportional to conductivity. Compared to MXene, the Ni-doped samples (Ni1-MX, Ni2-MX, Ni3-MX, Ni4-MX, and Ni5-MX) exhibit a substantial increase in dielectric polarization loss (Fig. 5h). Among them, Ni3-MX demonstrates the highest polarization loss, indicating that the introduction of electron localization significantly enhances the dielectric polarization capacity of the MXene matrix. To elucidate the frequency-dependent dissipation characteristics of Ni3-MX composites, we conducted a systematic quantification of polarization and conductive loss (Fig. S15). The polarization loss contributions exhibit a progressive enhancement with increase in frequency, while conductive loss demonstrates a gradual attenuation. These dual dissipation pathways demonstrate comparable significance within the lower frequency spectrum (2–4 GHz). However, multiple polarization loss processes emerge as the predominant factor, exhibiting greater influence than conductive loss (> 4 GHz). The evolution of Ni species and their hybrid interactions with MXene play a crucial role in improving polarization loss, highlighting its dominant contribution to electromagnetic wave attenuation.

Based on the above discussion, the variations in polarization loss follow a two-phase progression, as illustrated in Fig. 5j: an initial increase followed by a subsequent decrease. This trend corresponds to the transition from MXene to Ni1-MX, Ni2-MX, and Ni3-MX, followed by a decline in Ni4-MX and Ni5-MX. Initially, as nickel species are incorporated into MXene (Ni1-MX, Ni2-MX, and Ni3-MX), atomically dispersed Ni atoms coordinate with nitrogen species embedded in the MXene matrix, forming Ni–N–C structures that significantly enhance electromagnetic attenuation capabilities. The polarization loss curve suggests that dielectric polarization relaxation plays a key role in augmenting EMW absorption performance. The enhanced dielectric polarization observed in Ni2-MX is primarily attributed to dipolar polarization induced by the coordination of metal atoms with nitrogen species, leading to asymmetric electron redistribution. As a result, the dominant attenuation mechanism shifts from conduction loss in MXene to dipolar polarization in Ni1-MX and Ni2-MX. Furthermore, the embedded Ni atoms introduce room-temperature magnetism with a high saturation magnetization of 0.157 emu g^−1^ at 300 K, generating local magnetic moments that contribute to enhanced magnetic loss. As the nickel species evolve from isolated atoms to dispersed clusters, interfacial polarization induced by nanoscale interfaces further enhances dielectric polarization capacity. The newly introduced interfacial polarization contributes significantly to enhanced EMW absorption performance. Notably, Ni3-MX exhibits a significantly higher saturation magnetization of 0.459 emu g^−1^ at 300 K. The presence of magnetic exchange coupling between Ni sub-nanoclusters increases susceptibility to thermal disturbances, resulting in an anomalous increase in the coercivity (Hc) of Ni3-MX [52]. However, as the evolution progresses from clusters to larger nanoparticles, both conduction and polarization losses in Ni4-MX and Ni5-MX gradually diminish, leading to reduced absorption performance.

## Conclusion

This study introduces an electron localization-mediated strategy for tuning microwave absorption and successfully fabricates Ni-NC/MXene architectures with tailored electron confinement. The interaction between nickel nanoclusters and the MXene matrix induces localized charge redistribution, enhancing dipole polarization and dielectric loss. This mechanism enables precise control over the absorption band, achieving an outstanding RL_min_ of − 54 dB and an EAB of 6.8 GHz, with over 89% frequency coverage in the 2–18 GHz range. DFT calculations confirm that Ni species modulate the electronic structure, promoting interfacial polarization and optimizing electromagnetic dissipation. The transition from atomic-scale Ni doping to sub-nanocluster formation shifts the dominant loss mechanism from conduction loss in MXene to enhanced dipole polarization. Additionally, Ni sub-nanoclusters improve magnetic exchange coupling, further boosting microwave absorption efficiency. CST simulations validate significant RCS reduction, underscoring the potential of Ni-NC/MXene for stealth and electromagnetic wave attenuation applications. This work not only advances the understanding of electromagnetic loss mechanisms at the atomic level but also provides a robust strategy for designing high-performance microwave absorbers for next-generation stealth, telecommunications, and electromagnetic compatibility systems.

## Supplementary Information

Below is the link to the electronic supplementary material.

Supplementary file1 (DOCX 2406 KB)
