# Supplementary material for: Metal–Support Interaction Induced Electron Localization in Rationally Designed Metal Sites Anchored MXene Enables Boosted Electromagnetic Wave Attenuation
Source: Nanomicro Lett. 2025 Jun 23;17:309. doi: 10.1007/s40820-025-01819-9 (PMC12185853; doi:10.1007/s40820-025-01819-9)
Supplement: Supplementary file 1 — Supplementary file1 (DOCX 2406 KB) [file 40820_2025_1819_MOESM1_ESM.docx]

Supporting Information for

**Metal-Support Interaction Induced Electron Localization in Rationally Designed Metal Sites Anchored MXene Enables Boosted** **Electromagnetic Wave Attenuation**

Xiao Wang^1#^, Gaolei Dong^2#^, Fei Pan^1^, Cong Lin^2^, Bin Yuan^1^, Yang Yang^1^*, Wei Lu^1^*

^1^Shanghai Key Lab of D&A for Metal-Functional Materials, School of Materials Science & Engineering, Tongji University, Shanghai 201804, People’s Republic of China

^2^College of Materials Science and Engineering, Fuzhou University, Fuzhou 350108, People’s Republic of China

# Xiao Wang and Gaolei Dong contributed equally to this work.

*Corresponding authors. E-mail: [yangyang_@tongji.edu.cn](mailto:yangyang_@tongji.edu.cn) (Yang Yang); [weilu@tongji.edu.cn](mailto:weilu@tongji.edu.cn) (Wei Lu)

**S1 Corresponding formula in manuscript**

One Debye relaxation process corresponds to one semicircle (Cole-Cole semicircle) in accordance with Debye theory:

$\left( \varepsilon'-\frac{\varepsilon_{s}+\varepsilon_{\infty}}{2} \right)^{2}+\left( \varepsilon" \right)^{2}=\left( \frac{\varepsilon_{s}-\varepsilon_{\infty}}{2} \right)^{2}$ (S1)

$\varepsilon_{s}$ and $\varepsilon_{\infty}$ stand for the static dielectric constant, the dielectric constant at infinite frequency, respectively.

$∁_{0}={\mu"(\mu')}^{-2} f^{-1}$ (S2)

According to Debye theory, the relatively complex permittivity imaginary part (ε") could be divided into conduction loss (ε_c″_) and polarization loss (ε_p″_) as following equations:

$\varepsilon"=\frac{\omega\tau\left( \varepsilon_{s}-\varepsilon_{\infty} \right)}{1+\omega^{2}\tau^{2}}+\frac{\sigma}{\omega\varepsilon_{0}}=\varepsilon_{p}^{"}+\varepsilon_{c}^{"}$ (S3)

$\varepsilon_{c}^{"}=\frac{\sigma}{\omega\varepsilon_{0}}$ (S4)

where $\omega$ is angular frequency, $\varepsilon_{s}$ is static dielectric constant, 𝜏 is polarization relaxation time, $\varepsilon_{0}$is the permittivity of free space (8.85×10^-12^ *F/m*), $\varepsilon_{\infty}$ is relative permittivity at high frequency and 𝜎 is electrical conductivity.

Based on the transmission line theory, the reflection loss (RL) of absorbers is calculated by the complex permittivity ($\varepsilon_{r}$) and complex permeability ($\mu_{r}$) by the following equations:

$Z_{0}=\sqrt{\frac{\mu_{0}}{\varepsilon_{0}}}$ (S5)

$Z_{in}=Z_{0}\left( \frac{\mu_{r}}{\varepsilon_{r}} \right)^{\frac{1}{2}}tanh\left[ j\left( \frac{2\pi fd}{c} \right)\left( \varepsilon_{r}\mu_{r} \right)^{\frac{1}{2}} \right]$ (S6)

$RL(dB)=20 log\left| \frac{Z_{in}-Z_{0}}{Z_{in}+Z_{0}} \right|$ (S7)

where $Z_{in}$ and$Z_{0}$ are the input impedance of absorbent and impedance of free space, 𝑓, 𝑑 and 𝑐 stand for the frequency, thickness of absorbers and velocity of light, respectively.

The attenuation constant (α) can be calculated through:

$\alpha=\frac{\sqrt{2}\pi f}{c}\times\sqrt{\left( \mu^{''}\varepsilon^{''}-\mu'\varepsilon' \right)+\sqrt{{(\mu''\varepsilon'' - \mu'\varepsilon')}^{2}+{(\mu''\varepsilon'' + \mu'\varepsilon')}^{2}}}$ (S8)

The specific reflection loss (SRL) values can be described as:

S𝑅𝐿_min_=𝑅𝐿/ (𝑓𝑖𝑙𝑙𝑒𝑟𝑙𝑜𝑎𝑑𝑖𝑛𝑔 × 𝑙𝑎𝑦𝑒𝑟 𝑡h𝑖𝑐𝑘𝑛𝑒𝑠𝑠) (S9)

The Debye model can be used to express the dielectric polarization process in the following：

$\varepsilon^{'}=\varepsilon_{\infty}+\frac{\varepsilon_{s}-\varepsilon_{\infty}}{1+\left( 2\pi f\tau\right)^{2}}$ (S10)

${\varepsilon_{p}}^{''}=\varepsilon^{''}-\varepsilon_{c}^{"}=\frac{\varepsilon_{s}-\varepsilon_{\infty}}{1+\left( 2\pi f\tau\right)^{2}}\cdot\left( 2\pi f\tau\right)$ (S11)

where ε_s_, ε_∞_, f and τ are the static permittivity, the permittivity when the frequency is infinity, the frequency and the relaxation time, respectively.

$$t_{m}=\frac{n\lambda}{4}=\frac{nc}{4f_{m}\sqrt{\left| \varepsilon_{r}\parallel\mu_{r} \right|}} (S12)$$

where d_m_ and f_m_ stand for the matching thickness and the matching frequency.

**S2 Supplementary Figures**


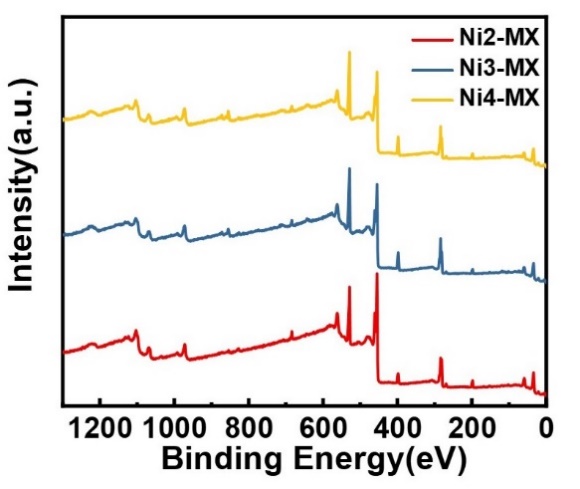


**Fig. S1** XPS survey scan spectra


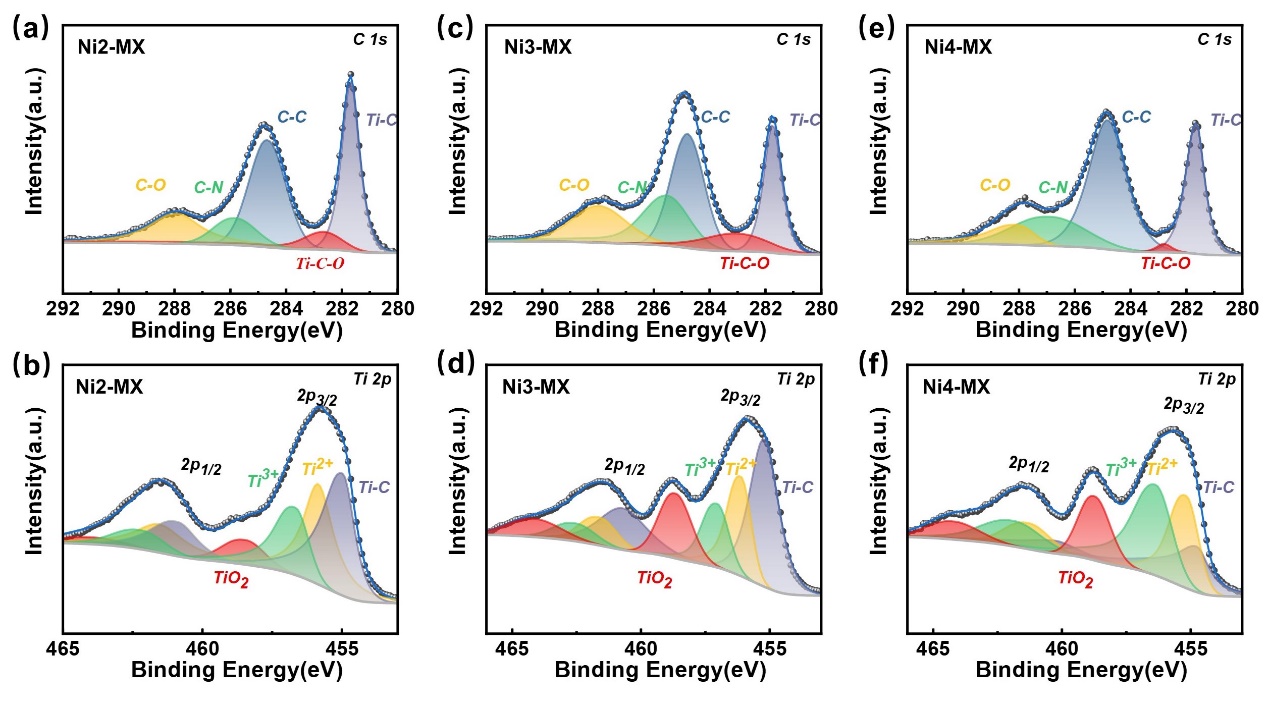


**Fig. S2** XPS survey scan spectra of Ti 2p and C1s


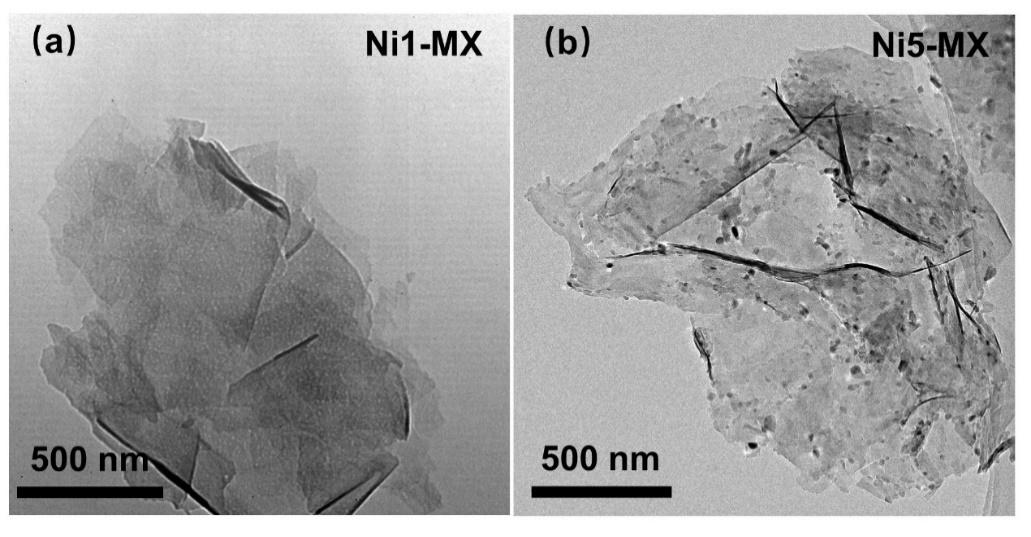


**Fig. S3** TEM image of Ni-MX

**Fig. S4** The size distribution diagram of Ni3-MX


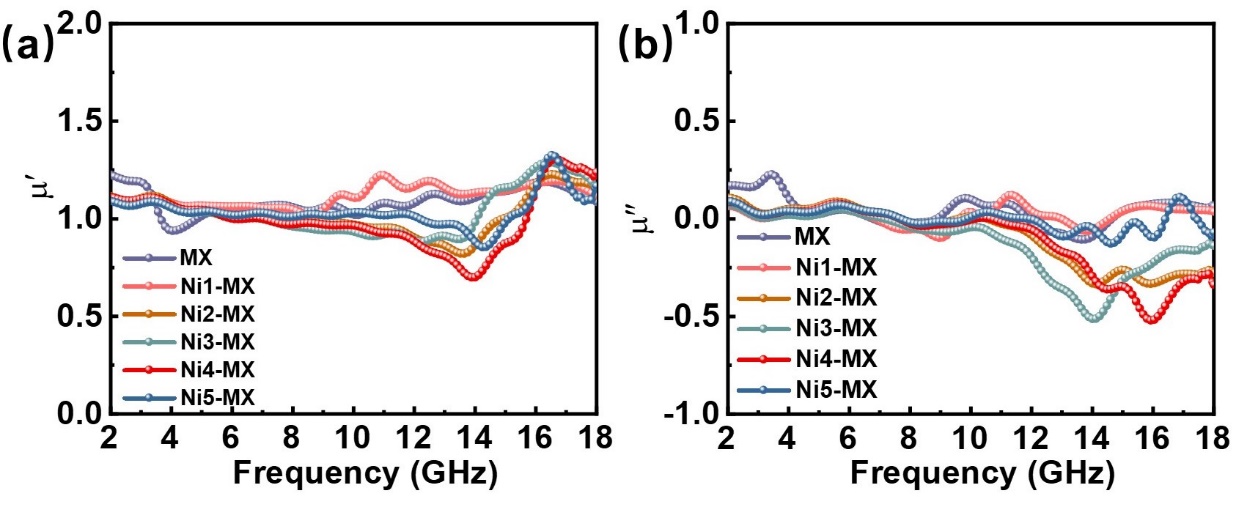


**Fig. S5** μʹ and μʺ of the Ni-MX

**
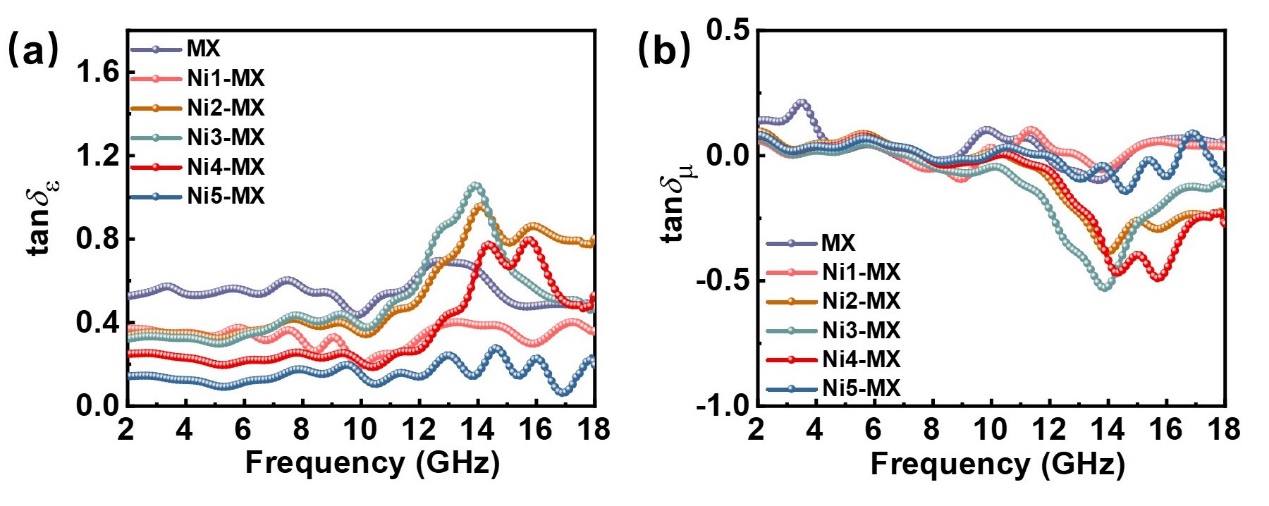
**

**Fig. S6** $\tan\delta_{\varepsilon}$ $\tan\delta_{\mu}$ of the Ni-MX


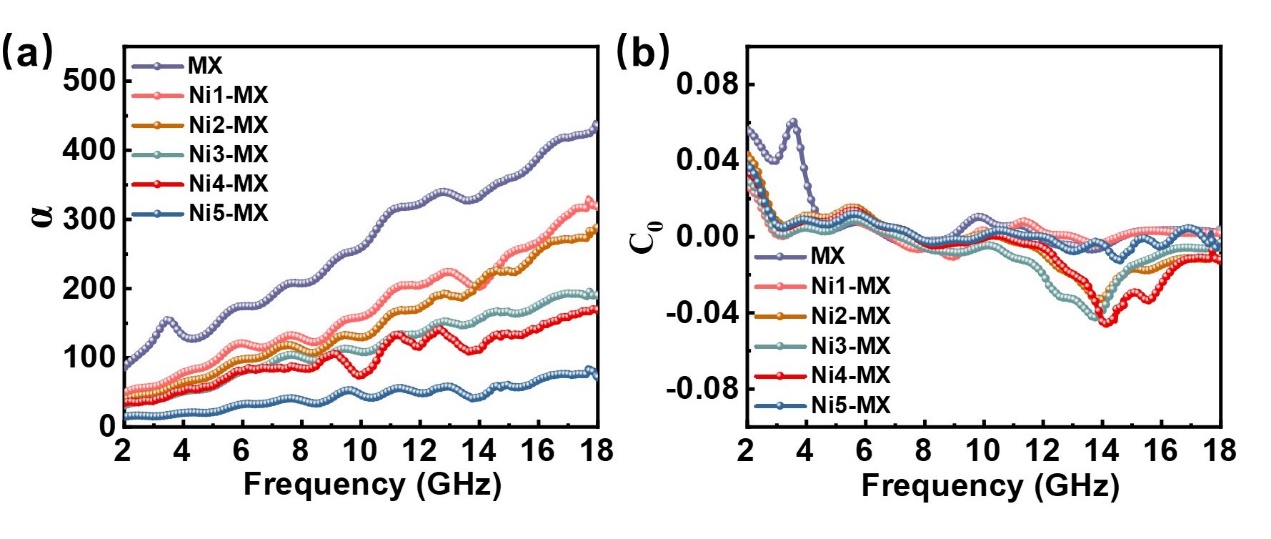


**Fig. S7** α and C_0_ of the he Ni-MX

**
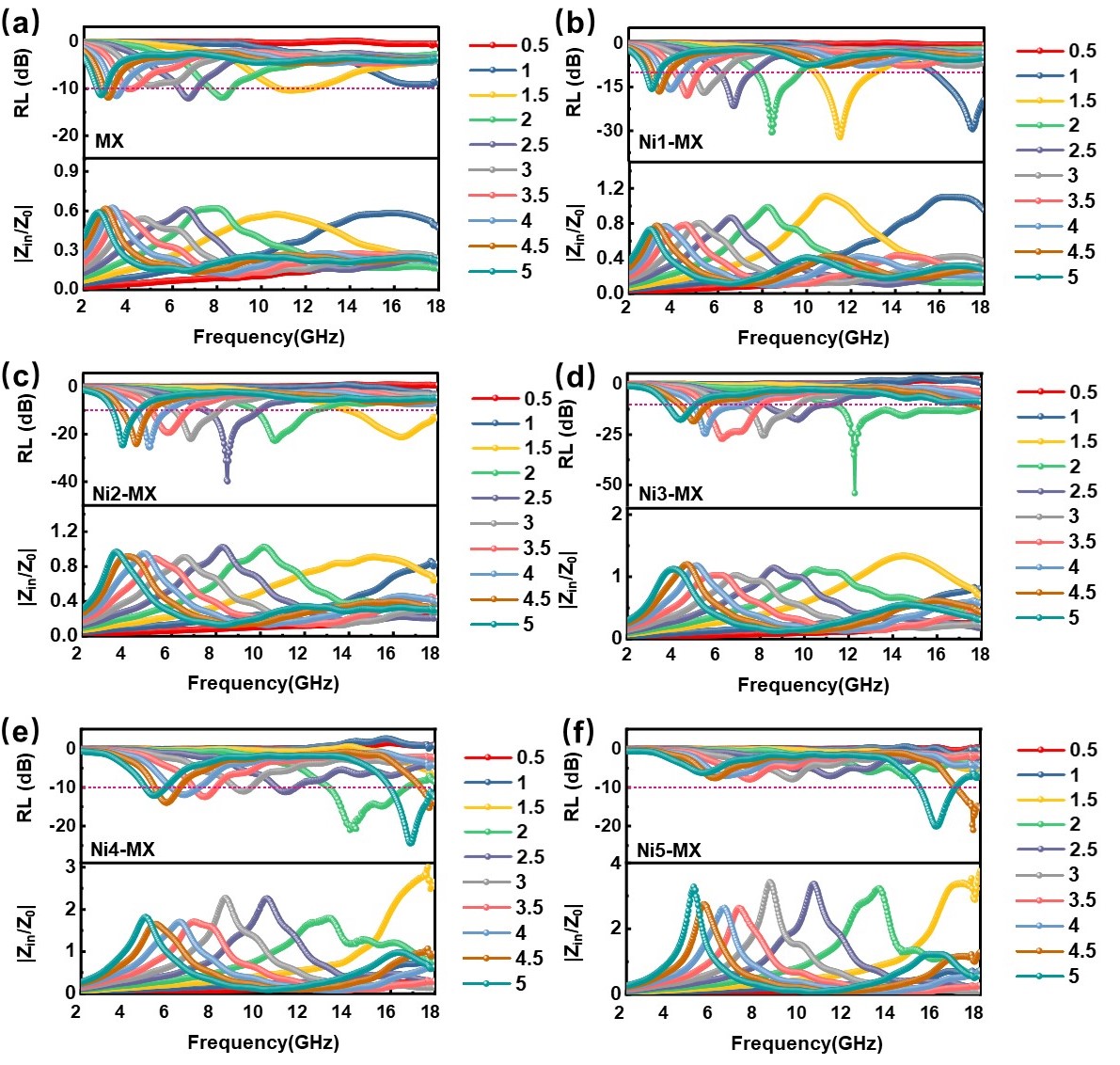
**

**Fig. S8** 2D RL and |Z_in_/Z_0_| of the he Ni-MX


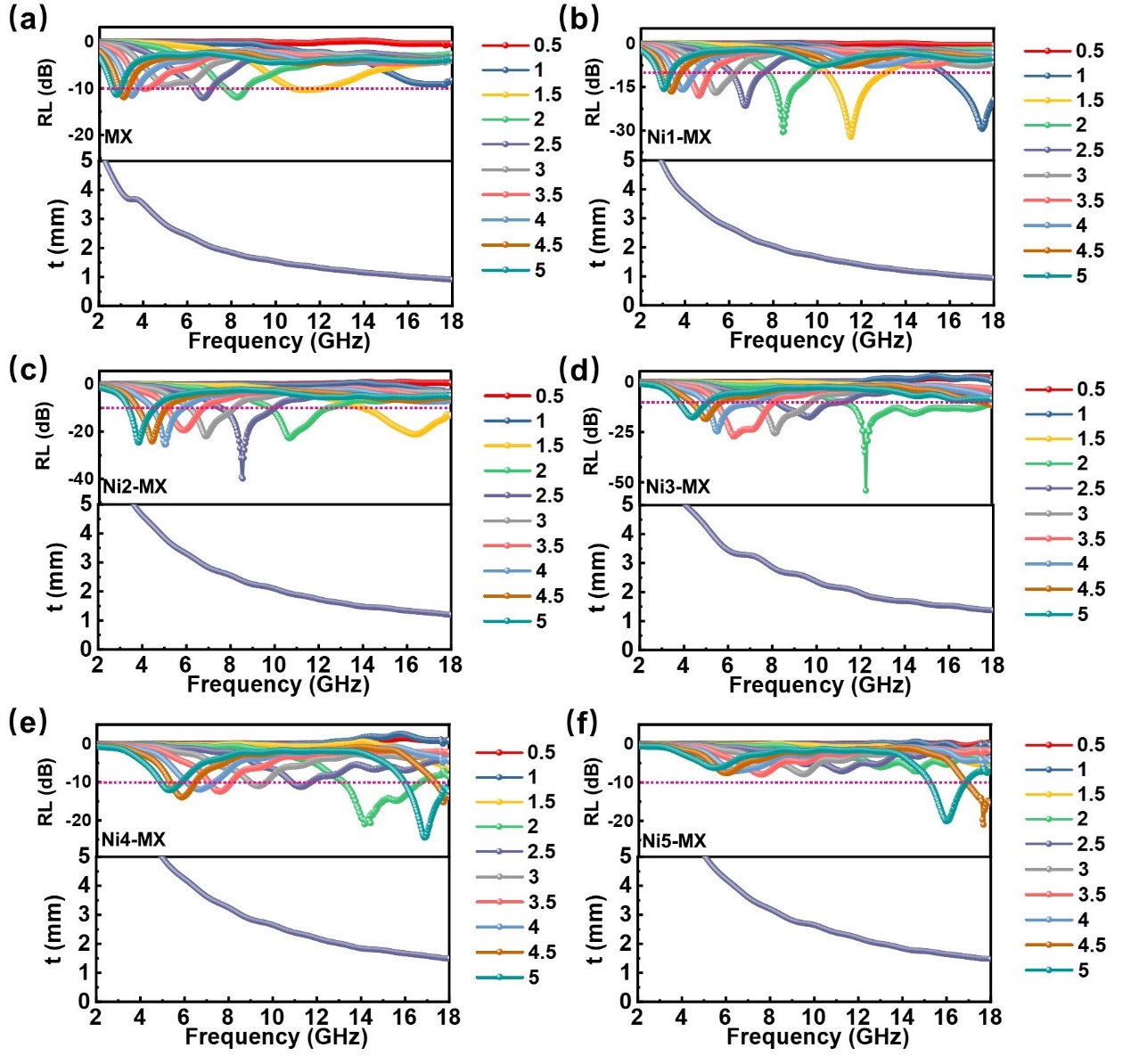


**Fig. S9** The quarter-wavelength thickness of Ni-MX


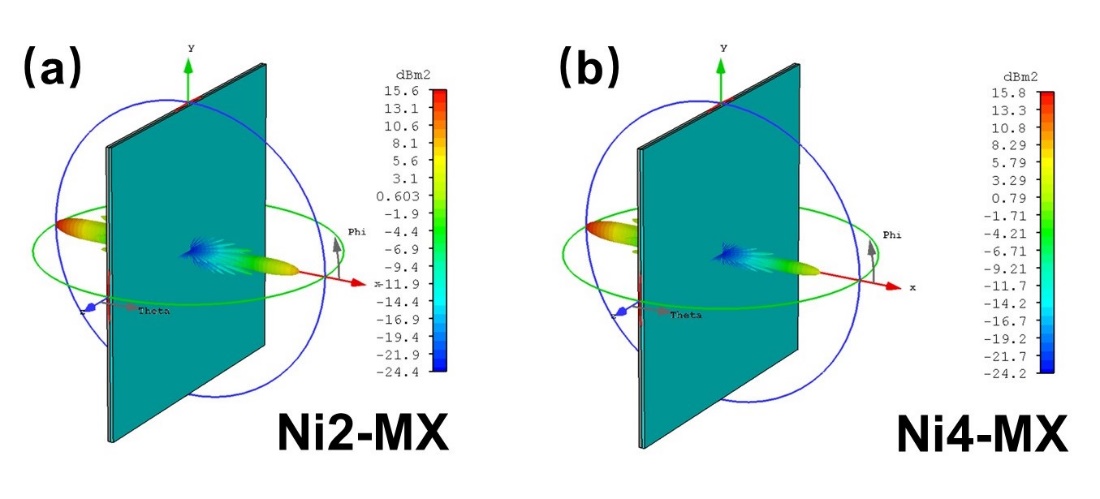


**Fig. S10** RCS of the he Ni-MX


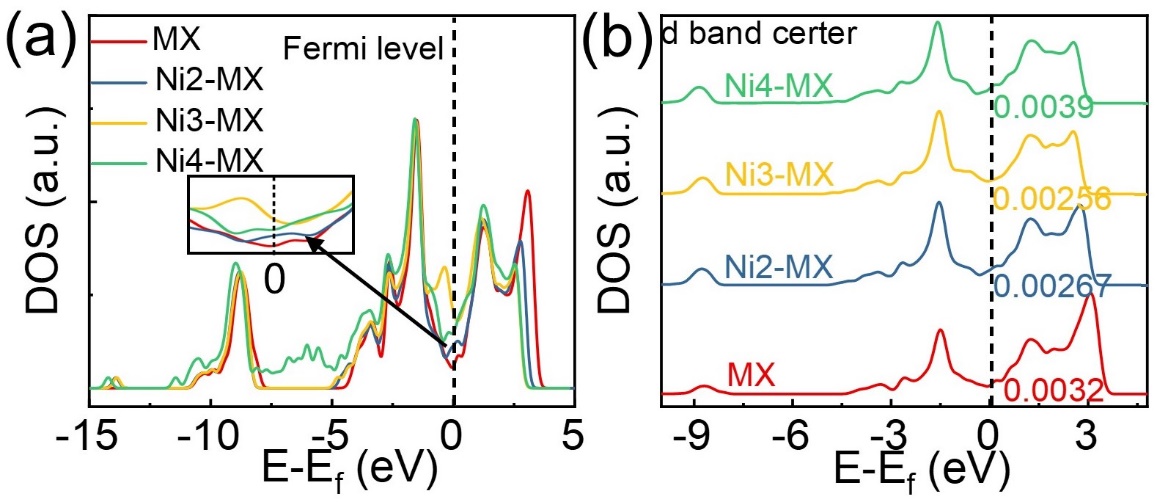


**Fig. S11** DOS of the Ni-MX

**Fig. S12** The work function of MX

**Fig. S13** Cole-Cole plot of the Ni-MX


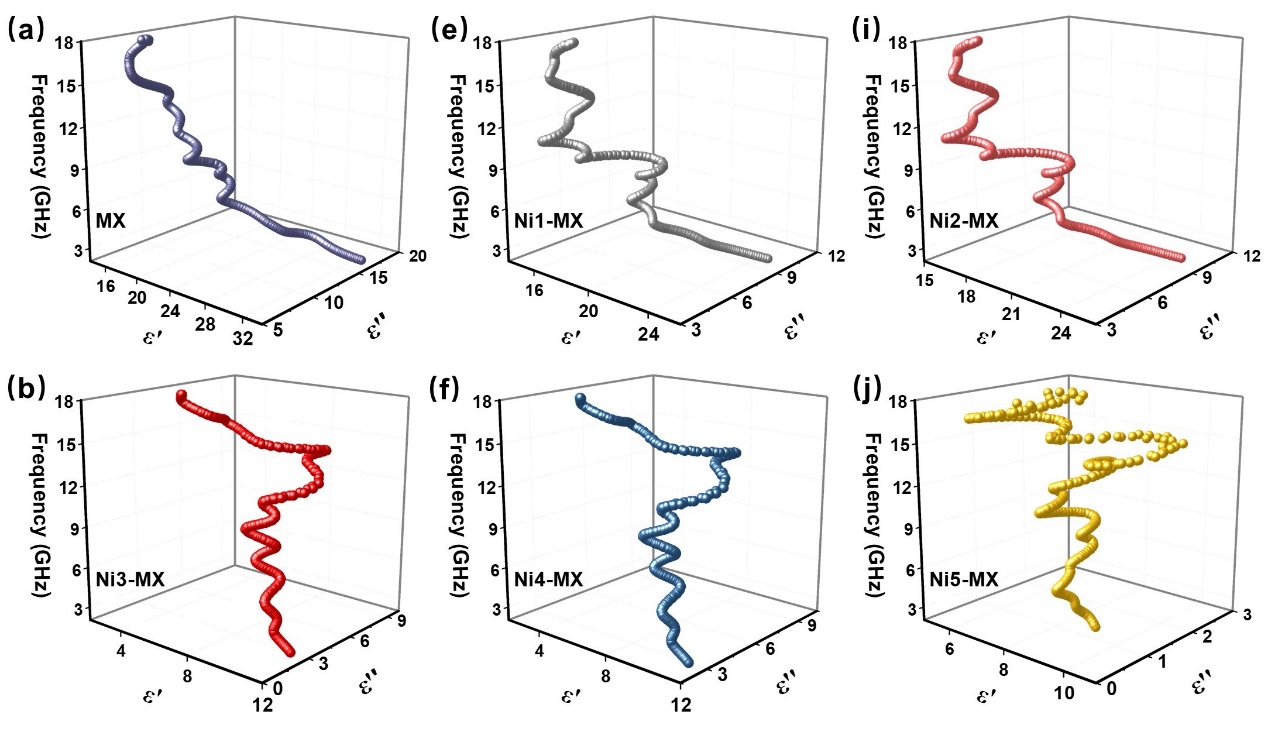


**Fig. S14** The 3D Cole-Cole plot of the Ni-MX

**Fig. S15** The quarter-wavelength thickness of Ni-MX
